# Face Aging by Explainable Conditional Adversarial Autoencoders

**DOI:** 10.3390/jimaging9050096

**Published:** 2023-05-10

**Authors:** Christos Korgialas, Evangelia Pantraki, Angeliki Bolari, Martha Sotiroudi, Constantine Kotropoulos

**Affiliations:** Department of Informatics, Faculty of Sciences, Aristotle University of Thessaloniki, 54124 Thessaloniki, Greece; ckorgial@csd.auth.gr (C.K.); epantrak@csd.auth.gr (E.P.); aggeliki.bolari@gmail.com (A.B.); marthass@csd.auth.gr (M.S.)

**Keywords:** explainable AI (xAI), generative adversarial networks (GANs), face aging

## Abstract

This paper deals with Generative Adversarial Networks (GANs) applied to face aging. An explainable face aging framework is proposed that builds on a well-known face aging approach, namely the Conditional Adversarial Autoencoder (CAAE). The proposed framework, namely, xAI-CAAE, couples CAAE with explainable Artificial Intelligence (xAI) methods, such as Saliency maps or Shapley additive explanations, to provide corrective feedback from the discriminator to the generator. xAI-guided training aims to supplement this feedback with explanations that provide a “reason” for the discriminator’s decision. Moreover, Local Interpretable Model-agnostic Explanations (LIME) are leveraged to provide explanations for the face areas that most influence the decision of a pre-trained age classifier. To the best of our knowledge, xAI methods are utilized in the context of face aging for the first time. A thorough qualitative and quantitative evaluation demonstrates that the incorporation of the xAI systems contributed significantly to the generation of more realistic age-progressed and regressed images.

## 1. Introduction

Face aging attempts to synthesize a person’s future facial appearance as they age or their past appearance as they regress. As individuals age, their facial features gradually and cumulatively change, resulting in certain common patterns, such as fine lines around the eyes and mouth and changes in skin texture. Although the effects of aging on facial appearance may differ from person to person, these patterns can be learned and applied to produce accurate simulations of how a face may age or become rejuvenated. The topic of face aging has triggered the interest of the research community due to its diverse range of applications, such as age-invariant face recognition for security purposes [1,2,3], entertainment-related applications [4], and the cosmetics industry [5].

Generative Adversarial Networks (GANs) [6] have played a vital role in generating realistic synthetic images for various applications. In [7], GANs were applied to face aging, which involved generating face images across different age groups. In [8], GANs were used to create synthetic images of traffic signs. Although GANs are highly effective in generating new data, they typically require a large and balanced dataset for proper training. Methods for addressing the class imbalance in classification tasks through the employment of GANs are explored in [9,10].

**Figure 1:**
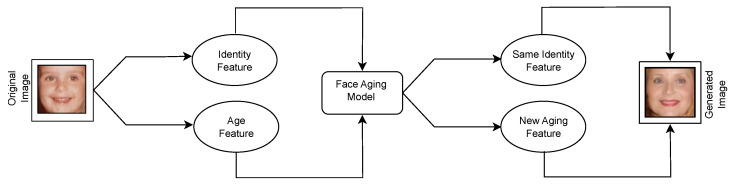
The face aging task.

This paper deals with GANs applied to facial images in the context of face aging. Figure 1 illustrates an example of face progression, where a young subject’s input face image is used to extract identity-related and age-related features fed into a face aging model. The model’s output is expected to preserve the identity-related features while incorporating aging patterns. In this paper, a state-of-the-art face aging GAN, namely, the Conditional Adversarial Autoencoder (CAAE) [7], is enriched with explanatory methods that provide insight into the discriminator’s decisions. The CAAE maps any face to a latent vector through a convolutional encoder. Then, the latent vector is projected to the face manifold conditional on age through a de-convolutional generator. More specifically, a facial image is faithfully reconstructed following the inversion process [11] by the generator. Simultaneous age progression and regression can be achieved by manipulating the age attribute.

The proposed framework integrates two explainable Artificial Intelligence (xAI) methods, namely, Saliency maps [12] and Shapley additive explanations (SHAP) [13], into the CAAE network. Saliency maps resort to the explanation matrix whose elements take values in the range [0,1], referring to pixels. A value close to zero indicates that the pixel has no impact on the classification decision made by the discriminator. Similarly, a value close to one implies the pixel significantly contributes. Here, we are interested in a modified gradient descent to update the generator’s weights. That is, the gradient of the discriminator’s decision with respect to the generator’s output (i.e., generated image) is used to derive the explanation matrix by taking the absolute value element-wise and scaling it in the range [0,1]. The explanation matrix is multiplied by a weight and added to the generator’s gradients with respect to the loss. The origin of the SHAP method is traced back to cooperative game theory. The payoff is the discriminator’s output, while the pixels of the images generated by the generator are treated as actors. Assume that one pixel is hidden from the rest of the pixels. The Shapley value is defined as the average marginal contribution of the withheld pixel in the discriminator’s output, which is calculated by averaging over all the different subsets that emerge along the path to form the grand coalition of pixels (i.e., the fully generated image) from the empty coalition of pixels. Let us refer to the CAAE coupled with xAI methods as xAI-CAAE. We explore whether improved corrective feedback from discriminator to generator occurs with the xAI-CAAE, improving its performance against CAAE.

A third xAI technique called Local Interpretable Model-agnostic Explanations (LIME) [14] is employed to determine the significant facial features contributing to face aging. By perturbing a set of facial images and collecting age classification decisions, LIME trains a regression model that approximates the age classifier in the local area of the training image. Consequently, if the explanation is consistent locally, it can be used to identify the significance of features.

The proposed framework incorporates xAI explanations by Saliency or SHAP into its training to enhance age progression and regression performed by GANs on facial images. LIME posterior explanations are derived as well. To the best of our knowledge, explainable methods are utilized in face aging employing several benchmark datasets for the first time. The main contributions of the paper are as follows:

1. **Explainable gradient-based methods are added to CAAE**: To add the reasoning of the discriminator’s decision into the generator’s training, the gradients of the generator are modified using either Saliency [12] or SHAP [13] explainable methods. The impact of xAI on the training process is explored by assigning different weights to the modified xAI gradients. The proposed xAI-CAAE is trained on a combination of images from the Cross-Age Celebrity Dataset (CACD) [15] and the UTKFace [7] datasets.
2. **Face aging assessment**: Both qualitative and quantitative evaluations of the generated facial images are conducted. The qualitative evaluation resorts to a visual inspection of the GAN-generated facial images. The quantitative assessment is two-fold: (i) The Fréchet Inception Distance (FID) [16] is computed for images produced by CAAE and xAI-CAAE applied to the FGNET dataset [17] to assess their visual quality; (ii) To estimate the age range of the generated age progressed and regressed FGNET images, the age estimation algorithm described in [18] is used. A thorough performance evaluation demonstrates the strengths of the proposed xAI-CAAE.
3. **Interpretation of age classification results using explainable occlusion-based methods**: To identify which facial areas are essential for age classification, experiments that utilize LIME’s posterior explanations are conducted on the Adience dataset [19].

The remainder of the paper is structured as follows. Related work and the proposed framework are surveyed in Section 2. Experimental evaluation is conducted in Section 3. Finally, Section 4 concludes the paper and recommends future work.

## 2. Materials and Methods

In Section 2.1, relevant research approaches on face aging are surveyed. Section 2.2 provides a brief overview of the xAI algorithms associated with the proposed method. Finally, the proposed xAI-CAAE is described in detail in Section 2.3.

### 2.1. Face Aging

Before the advent of deep learning, age progression and regression methods were primarily divided into physical model and prototype approaches. Physical model techniques [20,21] were focused on modeling the physical attributes of face aging, such as the alterations in hair, mouth, and skin texture, over time. These methods required a substantial amount of matched data and were time-consuming. Prototype-based approaches were focused on investigating the differences in facial images among different age classes to determine the aging patterns of each age class. Typically, these methods involved averaging the faces of individuals within the same age range to identify common features [22,23]. The face rejuvenation/progression process involved removing/adding textures that exhibited signs of aging, which was accomplished by applying a learned transformation across facial surfaces. Since this procedure produced smoothed facial images for each age class, maintaining identity information was challenging.

The success of GANs in image synthesis and translation tasks has led to remarkable progress in face aging techniques. The training process of GANs involves training two models simultaneously: the generator *G*, which learns to generate new samples that resemble the training samples and captures their distribution, and the discriminator *D*, which distinguishes the synthetically generated samples from the real training ones. Let the data distribution be $p_{data}\left(x\right)$, i.e., the training data x∼$p_{data}\left(x\right)$. If z is sampled from the distribution $p_{z}\left(z\right)$, *G* and *D* engage in the min-max game [6]

(1) $$\left\{G^{*},D^{*}\right\}=\underset{G}{\mathrm{argmin}}\;\underset{D}{\mathrm{argmax}}E_{x∼p_{data}\left(x\right)}\left[\log D(x)\right]+E_{z∼p_{z}\left(z\right)}\left[\log\left(1-D(G(z))\right)\right],$$

where E[·] denotes the expectation operator. To circumvent the prohibitive cost of longitudinal collection of multiple face images for each subject, GAN-based methods resort to unpaired face aging data for training and primarily focus on modeling mappings between image contents. It is crucial to enforce identity consistency to prevent matching ambiguities when trying to simulate the aging process in an unpaired training scenario. This way, key semantic conditional information of the input, such as the unique facial features of each individual, are maintained. It should be noted that most GAN-based face aging algorithms do not enforce constraints in regions that are particularly relevant to age changes. Still, the generator re-estimates the pixel at each spatial location of the synthetical image.

In [24], a GAN-based framework for Attribute-Aware Attentive Face Aging (A^3^GAN) was proposed. By integrating facial attribute vectors into the generator and discriminator, semantic conditional information from the input was employed to train the model to create elderly face images with attributes faithful to the corresponding input. An attention mechanism that limited the alterations to age-related regions improved the visual quality of the synthesized face images. Since wrinkles, eye bags, and laugh lines are represented mainly by local textures, a wavelet packet transform extracted features at several scales in the frequency space, increasing aging details.

The effects of aging within a specific age class are related to the effects of aging in neighboring age classes, as aging is a gradual and continuous process. Moreover, aging transformations between distant age classes are likely more drastic and intense than those between nearby age classes. In [25], face aging was addressed as an unsupervised image-to-image translation problem. The Pyramid Face Aging-GAN (PFA-GAN) was suggested in particular, which contains a pyramid weight-sharing method. Face aging effects are therefore learned hierarchically, beginning with the subtle changes necessary between neighboring age classes and progressing to the more conspicuous and drastic changes required between distant age classes. No paired examples showing how the person looks at the target age class were needed, alleviating a severe limitation of many face-aging approaches.

In [26], a framework was developed to simulate aging in three dimensions. The framework consisted of three components—a 3D estimator for vertices and textures, a texture-aging GAN, and a module for rendering 2D and 3D faces. The 3D vertex and texture estimator determined the spatial vertices and textures of the face. The GAN applied aging effects to the estimated texture map. Finally, the rendering module produced 2D or 3D faces using the vertex map and the aged facial texture map.

A GAN network termed Age Gap Reducer-Generative Adversarial Network was introduced in [27] to reduce the age gap between face images using automatic age estimation. The network took into account both the gender of the individual in the input image and the desired age group to which the input face needed to be adjusted. This enabled the network to either regress the input image to a younger age group or progress it to an older age group, depending on the desired outcome.

Face aging depends on accurate age estimation. Age estimation is a challenging task because it is affected by gender, race, and various intrinsic or extrinsic attributes. An age estimation architecture was developed in [28], which included three convolutional neural networks (CNNs) and two extreme learning machine structures. There is a lack of large and reliably annotated datasets for training deep neural networks to estimate age. Knowledge distillation was exploited for accurate age estimation in [29] to address this problem. In a nutshell, class probability vectors were derived by a large model comprising multiple CNNs applied to a reference annotated dataset. The resulting predictions were then exploited as target labels to train a smaller model on a face dataset without age annotations.

### 2.2. Basic Elements of Explainable AI

The paper’s main contribution is to enrich CAAE, described in Section 2.3, with explainable artificial intelligence (xAI) techniques by adding an explanation system into its training procedure. In the following, the basic xAI techniques are briefly reviewed.

Pixel and feature attribution techniques attempt to explain individual predictions by crediting each input feature according to how much it alters the prediction. Pixel attribution techniques are known by various names, one of which is Saliency maps. Typical examples of feature attribution techniques include SHAP and LIME. Input pixels, tabular data, or text are used as features. There are two sorts of attribution techniques:

1. **Gradient-based**: These techniques compute the gradient of the prediction (or classification score) concerning input features. The key distinction between various gradient-based techniques lies in the method they use to calculate the gradient.
2. **Occlusion-based**: These techniques, such as SHAP and LIME, manipulate image regions to produce model-agnostic explanations.

Both techniques provide explanations in the context of a Saliency map that is the same size as the input image or at least projected onto the input image. Each pixel is given a value, which can be interpreted as its importance for the prediction or categorization task.

#### 2.2.1. Saliency

Saliency maps [12] determine the significance of each feature in a given input for subsequent classification using a deep neural network inspired by how animals focus their attention. A nonlinear score function $S_{c}\left(x\right)$ is used to determine whether an image belongs to a particular class. This function is linearized using a first-order Taylor expansion around a specific image $x_{0}$

(2) $$S_{c}\left(x\right)\approx S_{c}\left(x_{0}\right)+\nabla_{x}S_{c}^{⊤}\left(x_{0}\right)\;\left(x-x_{0}\right).$$

It is seen that the first term represents the classification score when the input is not perturbed, while the gradient term weighs the impact of perturbations. By reshaping the gradient to a two-dimensional matrix (i.e., an image), the Saliency map M is produced. The Saliency map is frequently normalized. Accordingly, M will refer to the normalized Saliency map hereafter. If more than one channels are present in the input image, the maximum Saliency map across all channels is considered. One can create a visual representation by taking the absolute values of the elements in the Saliency map or by distinguishing between the negative and positive contributions. The process of computing the Saliency map is not costly, as it only necessitates a single back-propagation step and does not assume the existence of any further annotations beyond the labels employed during the initial model training.

#### 2.2.2. LIME

LIME [14] selectively activates or deactivates certain super-pixels in an image and then examines how these perturbations impact the predictions made by a classifier. To achieve this, LIME creates a synthetic neighborhood, denoted by N(x), around the input instance to be explained, denoted by $x\in R^{d}$, i.e.,

(3) $$N\left(x\right)=\left\{x_{j}|x_{j}=x+p_{j},\;\;p_{j}∼N\left(0,\Sigma \right)|j=1,2,\ldots ,H\right\},$$

where $p_{j}$ is a local perturbation, and $N\left(0,\Sigma \right)$ denotes the zero-mean multivariate normal distribution with diagonal covariance matrix Σ estimated from the training set. Let $x^{\prime }\in {\left\{0,1\right\}}^{d^{\prime }}$ denote a binary vector for the interpretable representation of x, i.e., a binary vector indicating the “presence” or “absence” of a super-pixel. Let $f:\;R^{d}\to R$ be the model to be explained. f(x) can be either a probability value or a binary indicator that indicates whether x belongs to a specific class. To learn a potentially interpretable linear model $g\left(x\right)=w_{g}^{⊤}\;x$, LIME samples instances $x^{\prime }$ from N(x) by randomly selecting non-zero elements. Given a perturbed sample $\zeta^{\prime }\in {\left\{0,1\right\}}^{d^{\prime }}$ for $d^{\prime }<d$ that contains the fraction of non-zero elements in $x^{\prime }$, the ridge regression method is used to learn g(x) by defining a locally weighted square loss function as

(4) $$L\left(f,g,\pi_{x}\right)=\sum_{x^{\prime },\;\zeta^{\prime }\in N\left(x\right)}\pi_{x}\left(x^{\prime }\right)\;{\left(f\left(x^{\prime }\right)-g\left(\zeta^{\prime }\right)\right)}^{2},$$

where $\pi_{x}\left(x^{\prime }\right)=\exp\left(-\frac{\left|\right|x^{\prime }-\zeta^{\prime }{\left|\right|}_{2}^{2}}{\varrho^{2}}\right)$ is an exponential kernel with width ϱ. Next, the function $g^{*}\left(x\right)$ is sought that minimizes

(5) $$g^{*}\left(x\right)=\underset{g}{\mathrm{argmin}}L\left(f,g,\pi_{x}\right)+\Omega \left(g\right),$$

where the minimization is performed over the set of linear models, and Ω(g) is a measure of interpretability (i.e., the number of non-zero weights for linear models). To solve the optimization problem (5), the least absolute shrinkage and selection operator (LASSO) is employed.

LIME utilizes a sequential feature selection [30,31] to fit multiple ridge regressors and select a subset of *F* features for the model *g* repeatedly. The kernel width used is $\varrho =\frac{3}{4}\sqrt{F}$, which is chosen during the process.

#### 2.2.3. SHAP

Shapley’s values, which came from cooperative game theory, are the cornerstone of SHAP [13]. In SHAP, features are considered actors that can potentially form coalitions to maximize future profits in a collaborative ecosystem. This approach has served as the foundation for several fields due to its adaptability. KernelSHAP (abbreviated as SHAP) is one of these branches.

SHAP approximates the original model with the surrogate model, chosen as a linear one. Let the original black-box model (i.e., GAN) be *f* and the surrogate model be denoted as *g*. Moreover, let $z^{\prime }$ be a reduced vector of ones and zeros that enables or disables certain features of z, also known as the coalition vector. If *m* denotes the size of z and ξ is the number of ones in $z^{\prime }$, SHAP’s loss function is defined as

(6) $$L\left(f,g,\pi_{x}\right)=\sum_{z^{\prime }\in Z}{\left[f\left(h_{x}\left(z^{\prime }\right)\right)-g\left(z^{\prime }\right)\right]}^{2}\pi_{x}\left(z^{\prime }\right),$$

where $h_{x}\left(z^{\prime }\right)$ reshapes the reduced vector $z^{\prime }\in R^{\xi }$ to $R^{m}$ and *Z* denotes the set of all possible reduced vectors to be generated by taking subsets of features from $z\in R^{m}$. The SHAP kernel $\pi_{z^{\prime }}$ in (6) is given by:

(7) $$\pi_{x}\left(z^{\prime }\right)={\left[\frac{m!}{\xi !\;(m-\xi )!}\right]}^{-1}.$$

The SHAP kernel is critical for giving tiny or big coalitions greater weight than coalitions that merely combine half of the traits (or close to it). The idea behind these behaviors is that we can learn more about individual features if we can analyze them separately (small coalitions) or if we have nearly all features except one (big coalitions).

The SHAP method is a reliable approach that can yield results equivalent to, if not better than, that of LIME. It also draws on well-established notions such as Shapley values, game theory, and LIME’s intuitive reasoning. However, similar to other permutation-based methods, SHAP has the issue of creating unrealistic data points by replacing missing attributes with random ones, which might lead to exaggerated interpretations.

#### 2.2.4. Overview of xAI-Enhanced Approaches

This research parallels other initiatives to equip GANs with explainable techniques. In [32], a study was conducted to investigate the similarity of the inner structure of CNN-based generators employed in CycleGAN. The CycleGAN was previously used for face aging in [33]. The study introduced a cross-GAN filter similarity index to analyze the similarity of CNN filters across different GANs. Another explainable methodology called GAN-based Model EXplainability (GANMEX) was developed in [34] by incorporating the classifier into the GAN to generate one versus many explanations. By using Principal Component Analysis in the latent feature space, important latent directions were identified, which enabled a large number of interpretable controls through layer-wise perturbation [35]. In [36], an xAI-enhanced version of a baseline machine learning model is proposed that is proved to outperform the original model in terms of interpretability and classification accuracy. The SHAP technique was used to extract high-contributed features that led to more accurate identification of vegetation pixels in [37].

### 2.3. Proposed Workflow

**Figure 2:**
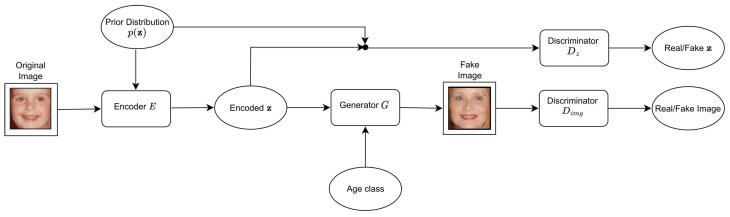
The CAAE architecture.

The baseline CAAE network is depicted in Figure 2. The CAAE model includes the encoder *E*, the generator *G*, and two discriminators, namely, $D_{img}$ and $D_{z}$. *G* is implemented as an autoencoder. Given an input face image, the encoder *E* generates an encoded z (also known as a latent vector), preserving the high-level personal feature of the input face. The generator *G* uses the encoded z and the target age information as a label to generate a facial image conditioned on the age. Two discriminator networks are imposed on the encoder *E* and the generator *G*, respectively. $D_{z}$ regularizes z to be uniformly distributed to smooth the age transformation. $D_{img}$ enforces *G* to generate photo-realistic and plausible faces for arbitrary z and age label. The objective function contains three terms: (1) the $\ell_{2}$ norm of the reconstruction error between the input image and the generated image by the generator plus the total variation loss of the generated image to remove ghosting artifacts; (2) the min-max objective function to train the encoder and $D_{z}$; and (3) the min-max function to train $D_{img}$. Let x denote the input face image, *l* refer to an age label, and z be the encoded variable in the output of the encoder E(x)=z. Moreover, let L(·,·) and TV(·) be the $\ell_{2}$ error norm and the total variation, respectively. If $p_{data}\left(x\right)$ is the distribution of the training face images, p(z) denotes the prior distribution, and $z^{\prime }∼p\left(z\right)$ implies random sampling from the prior distribution, the objective function optimized by CAAE is defined in (8), i.e.,

(8) $$\begin{array}{cc} \; & \left\{E^{*},G^{*},D_{z}^{*},D_{img}^{*}\right\}=\underset{E,G}{\mathrm{argmin}}\underset{D_{z},D_{img}}{\mathrm{argmax}}\{\lambda \;L\left(x,G(E(x),l)\right)+\gamma \;TV\left(G(E(x),l)\right)\\ \; & +E_{z^{\prime }∼p_{z}\left(z\right)}\left[\log\;D_{z}\left(z^{\prime }\right)\right]+E_{x∼p_{data}\left(x\right)}\left[1-\log\;D_{z}\left(E\left(x\right)\right)\right]\\ \; & +E_{x,l∼p_{data}\left(x,l\right)}\left[\log\;D_{img}\left(x,l\right)\right]+E_{x,l∼p_{data}\left(x,l\right)}\left[\log\;\left(1-D_{img}\left(G\left(E\left(x\right),l\right)\right)\right)\right]\}, \end{array}$$

where the coefficients λ and γ balance the smoothness and high-resolution terms.

CAAE is capable of generating highly realistic face images with both regressive and progressive effects. Unlike other methods, CAAE does not need paired examples of data for training or labeled faces in the testing data, making it more flexible. The separation of age and personality information in the latent space of z results in maintained individual personalities and the elimination of any ghosting artifacts. Finally, CAAE is not influenced by alterations in pose, emotions, or occlusion.

**Figure 3:**
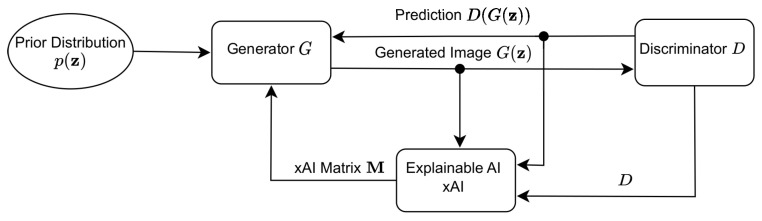
xAI-GAN system architecture.

Another network termed xAI-GAN refers to the technique that aims to enhance GANs to provide better synthetic or reconstructed images through the incorporation of an xAI system [38]. In this way, corrective explanatory feedback is provided during training from the discriminator to the generator. Figure 3 depicts the system architecture of xAI-GAN.

Given a noise sample z from a noise distribution, the untrained generator *G* creates an image G(z) that is then fed to discriminator *D*. The output of the discriminator D(G(z)), the generated image G(z), and the discriminator network *D* are channeled to the xAI system seeking an explanation of the loss incurred by the synthetic image G(z). The general idea behind the xAI-guided training process of a GAN is that the xAI system works as a guide. The xAI system acts by structuring the gradient descent in such a manner that generator training is focused on the most essential input features that the discriminator identifies.

**Figure 4:**
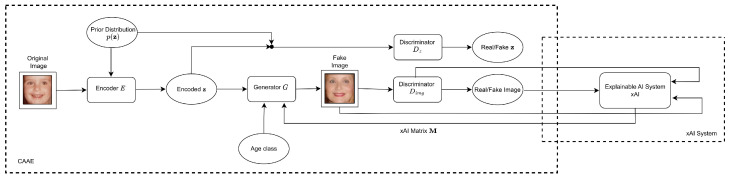
Proposed xAI-CAAE architecture.

The proposed framework follows the network structure in CAAE, which includes an encoder *E* that converts RGB images to latent vectors z, a generator *G* that converts z to RGB images, a discriminator $D_{z}$ that enforces a uniform distribution on the encoder’s output, and a discriminator $D_{img}$ that ensures the generator creates realistic images. CAAE incorporates two discriminators to improve the realistic properties of the generated facial images. The proposed xAI-CAAE aims to leverage xAI systems to strengthen and enrich the age progression and regression accomplished by CAAE. In CAAE, the discriminator offers feedback to the generator using a single loss value per generated image. The aim of xAI-guided training is to enhance this feedback by providing the xAI system’s “reasoning” for the discriminator’s decision. The architecture of xAI-CAAE is depicted in Figure 4.

In xAI-CAAE, a modified gradient descent generator training process is established so that generator training focuses on the most significant features for the discriminator’s prediction. An xAI system employs a score function $S_{c}$ to determine the explanation matrix $M=S_{c}\left(G\left(z\right)\right)$ after propagating the loss through the discriminator $D_{img}$ to find $\Delta_{G(z)}$. The matrix M, which refers to pixels, is made up of real numbers in the range [0, 1], with greater values indicating more important features for the discriminator’s prediction. More specifically, in Saliency maps, if the pixel is given a value of 0 or near 0 in M, the pixel under consideration does not influence the discriminator’s classification decision. On the contrary, the pixel is considered extremely essential if given a value of 1 or near 1. These values, when approaching 1, also show high-quality classification by the discriminator. In SHAP, the Shapley value can be described as the mean incremental impact of each pixel that is excluded in the discriminator’s output. This is computed by taking an average of all the different combinations of pixels that form the complete image, starting from an empty set of pixels. Accordingly, the explanation matrix M allows us to concentrate the learning process on the most important qualities, regardless of whether they were favorable or harmful to the classification.

The proposed framework utilizes M to update the generator’s weights in a modified gradient descent method. In CAAE, the adjustment of generator weights typically involves calculating the gradient of the generator’s output with respect to the loss and then applying the chain rule. This method is enhanced by first computing the explanation matrix M and then multiplying it by the gradient of the generator’s output with respect to the loss. The explanation matrix M is used to mask the latter gradient and the pixels that contributed to the discriminator’s classification. As described, the modified gradient $\Delta_{G(z)}$ is obtained by taking the Hadamard product (element-wise multiplication) between $\Delta_{G(z)}$ and M, denoted as $\Delta_{G(z)}⊙M$, which serves as a mask for G(z) and restricts the gradient to the most significant elements. Finally, the generator’s gradients $\Delta_{G(z)}^{\prime }$ are computed using the modified gradient as

(9) $$\Delta_{G(z)}^{\prime }=\Delta_{G(z)}+\theta \;\Delta_{G(z)}⊙M,$$

where θ is a parameter that determines the degree to which the xAI system affects the original gradients.

## 3. Results

Here, experimental findings for the proposed framework are disclosed. In Section 3.1, implementation details are provided for xAI-CAAE. Section 3.2 discusses the datasets utilized in the experimental evaluation. In Section 3.3, the qualitative evaluation for xAI-CAAE is described. A thorough quantitative evaluation for xAI-CAAE is discussed in Section 3.4, while important facial features for face aging are investigated in Section 3.5 using the LIME explanation system.

### 3.1. Implementation Details

The implementation of the proposed xAI-CAAE framework is based on the publicly available code for CAAE [7] (https://github.com/mattans/AgeProgression/tree/v1.0.0, accessed on 8 May 2023) and xAI-GAN [38] (https://github.com/explainable-gan/XAIGAN, accessed on 8 May 2023). xAI-CAAE is implemented using the Pytorch 1.2.0 [39] library. The Captum 0.4.0 [40] library is also used to implement the Saliency and SHAP explanations. For LIME, we used Lime 0.2.0.1.

The proposed xAI-CAAE, based on the setup described in [7], takes as input images of size 128×128×3. Both the encoder *E* and the generator *G* use a kernel size of 5×5. The encoder consists of five convolutional layers, each of which is followed by a ReLU activation function, and a fully connected layer, which is followed by the hyperbolic tangent activation function. The generator consists of a linear layer and seven de-convolution layers, each of which is followed by the ReLU activation function. The discriminator $D_{z}$ is composed of four fully connected layers. The discriminator $D_{img}$ uses a kernel size of 2×2 and comprises four convolutional layers, each of which is followed by batch normalization and a ReLU activation function, as well as two fully connected layers followed by the sigmoid activation function.

The input image intensities are normalized within the range [−1, 1], and then they are fed to the encoder *E*. The output of *E* is represented by the encoded vector z, whose elements are also limited within the range [−1, 1] due to the hyperbolic tangent activation function. Subsequently, the age and gender information is transformed into a one-hot vector, which is also constrained to the range [−1, 1] (instead of the usual range [0, 1]), and then concatenated with the encoded vector z. This concatenated vector is used as input for the generator *G*, which generates an image with intensities within the range [−1, 1] due to the hyperbolic tangent activation function. During the training process, the mini-batch size is set to 64, and the network’s four blocks (*E*, *G*, $D_{z}$, and $D_{img}$) are updated accordingly. The Adaptive Moment Estimation (ADAM) optimizer [41] with a learning rate of 0.0002, $\beta_{1}=0.9$, $\beta_{2}=0.999$, and weight decay of $10^{-5}$ is employed. The network is trained for 200 epochs.

In order to integrate the xAI system with CAAE, a sigmoid activation layer is added to $D_{img}$, which provides predictions within the range [0, 1]. By doing so, the prediction of $D_{img}$ for generated images is in the [0, 1] range, allowing images with high prediction values to be masked. The explanation matrix derived from any of the xAI systems undergoes two processing steps by taking the absolute value of the elements of M and then normalizing the resulting absolute values. The processed explanation matrix M is utilized as a mask for *G*. The autograd package [42] of Pytorch, which supports automatic tensor differentiation, is utilized in the xAI implementation. To modify the gradients of the generator using the explanation matrix M and adjust the backpropagation method, the register backward hook method is used, as described in Section 2.3. After half of the training epochs, xAI-guided gradient descent is utilized, as per [38].

### 3.2. Datasets

The proposed xAI-CAAE framework is trained on a set of images that were collected from the CACD [15] and the UTKFace [7] dataset. This set of images was collected and used to train the face aging approach in [25]. It includes 21,267 face images distributed to seven age classes: 0–10, 11–18, 19–29, 30–39, 40–49, 50–59, and 60+ years old (the oldest person is 80 years old). The same split to age classes has been considered in many facing approaches [25,43]. Approximately the same number of images belongs to each age class. Each gender is equally distributed in each class.

The FGNET aging dataset [17] is employed for testing xAI-CAAE. FGNET comprises 1002 face images of 82 different subjects, whose age varies from 0 to 69 years. FGNET is a dataset frequently used in facial aging research [7,44,45].

The Adience dataset [19] is a collection of images collected from the social network Flickr. The dataset comprises 26,580 images distributed to eight age classes: 0–2, 4–6, 8–13, 15–20, 25–32, 38–43, 48–53, and 60+. The dataset is very challenging for age and gender classification due to the unconstrained, real-life capturing conditions of its images. This dataset is used to investigate which facial regions are important for age classification by leveraging LIME explanations, as described in Section 3.5.

### 3.3. Qualitative Evaluation of xAI-CAAE

**Figure 5:**
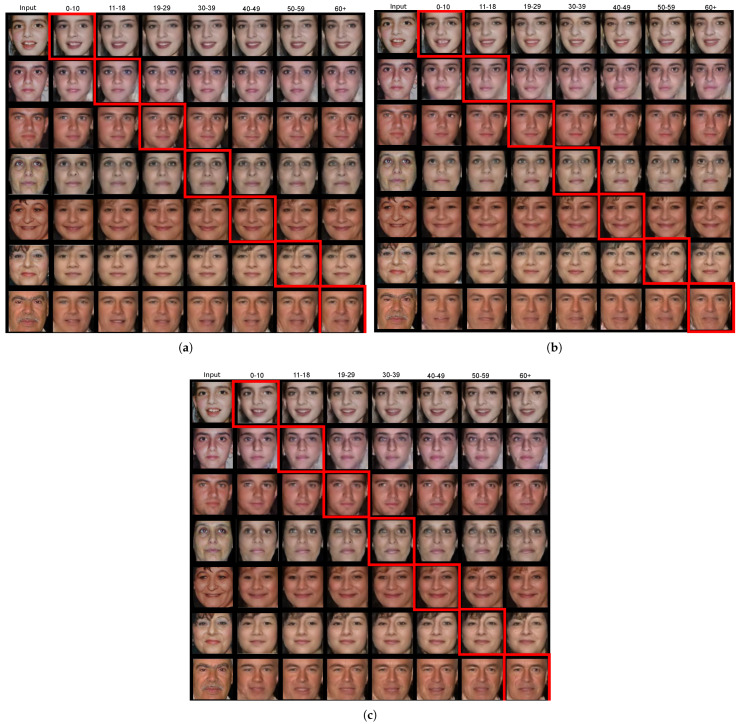
Age progression and regression results obtained by xAI-CAAE for the size of latent vector z equal to 100 and θ=0.2, when using (**a**) Saliency and (**b**) SHAP xAI systems to sample images of FGNET. The results in (**c**) are obtained by the original CAAE without incorporating any xAI system for the same size of the latent vector z. The first column depicts input images, and the rest of the columns depict the age-progressed and regressed images. The red boxes indicate the generated images belonging to each input image’s ground truth age class.

**Figure 6:**
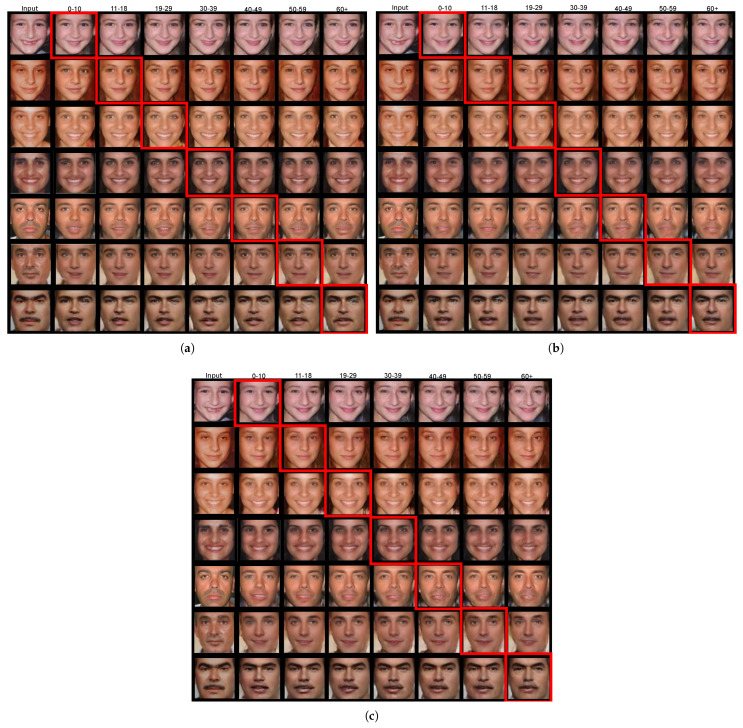
Age progression and regression results obtained by xAI-CAAE for the size of latent vector z equal to 100 and θ=0.5, when using (**a**) Saliency and (**b**) SHAP. The results in (**c**) are obtained by the original CAAE without incorporating any xAI system for the same size of the latent vector z. The first column depicts input faces, and the rest of the columns depict the age-progressed and regressed generated images. The red boxes indicate the generated images belonging to each input image’s ground truth age class.

The proposed framework generated age progression and regression results on the FGNET dataset using two xAI systems, Saliency and SHAP. These results are depicted in Figure 5a and Figure 5b, respectively. Figure 5c depicts comparative results for the original CAAE. The images in the first column of each figure illustrate the sample FGNET images from each age class, while the rest of the columns illustrate the generated images in each age class. The ground truth age class of each image is indicated by a red box. In the experiments shown in Figure 5, the xAI-CAAE parameters were set to a z size of 100 and θ=0.2 in (9). In Figure 6, results with a z size of 100 and θ=0.5 in (9) are depicted.

**Comparison between xAI-CAAE and CAAE**. As can be observed in Figure 5, xAI-CAAE yields plausible and satisfying age progression and regression results. The images generated by xAI-CAAE are more realistic, with fewer distortions compared to the images generated by CAAE. For example, in the second row of Figure 5a–c, age-progressed and regressed images generated by xAI-CAAE using either Saliency or SHAP are notably more pleasing compared with the images generated by CAAE, which produced blurring in the image, especially around the area of the eyes. The same can be observed for the images in the fourth row of Figure 5a–c, where xAI-CAAE using either Saliency or SHAP generates more realistic facial images compared to the images generated by CAAE that include eye distortions, especially for age progression (columns 6–8). Similar observations can be made for the age progression and regression results in Figure 6a–c. For example, inspecting the images in the second, third, and fifth rows shows that the proposed framework produces more realistic images with fewer artifacts compared to the images generated by CAAE. The qualitative inspection reveals the advantages of using xAI techniques in the CAAE network.

**Comparison between different xAI techniques in xAI-CAAE**. Comparing the results achieved by xAI-CAAE in Figure 5a,b and Figure 6a,b, xAI-CAAE with Saliency achieves competitive performance to xAI-CAAE using SHAP. In some cases (e.g., the fifth and sixth rows in Figure 5a,b), the images generated by xAI-CAAE using Saliency are sharper and more detailed compared to the ones generated by xAI-CAAE using SHAP. However, in the same rows, the images generated by xAI-CAAE using SHAP more realistically represent the process of face aging, especially in the older age classes. More specifically, the SHAP method seems to render with greater success the characteristics of face age progression, i.e., faces with wrinkles around the eyes and mouth (see column 8 in the sixth row of Figure 5a,b).

### 3.4. Quantitative Evaluation of xAI-CAAE

In this Section, xAI-CAAE is evaluated using the quantitative evaluation metrics described in Section 3.4.1.

#### 3.4.1. Evaluation Metrics

**Fréchet Inception Distance**. To evaluate the quality of images generated by CAAE and xAI-CAAE, the Fréchet Inception Distance (FID) is employed. This metric has been shown to align with human perception of image quality [16]. FID works by mapping a set of images to a feature space defined by a specific layer of the Inception model. The layer’s activation values are used to estimate statistics such as the mean vector and the covariance matrix, which are employed to create a multi-dimensional Gaussian distribution. Finally, the Fréchet Distance between the distributions estimated from the real and generated images is calculated and denoted by the FID score. When the FID score is lower, it signifies that the generated images closely resemble the real images, indicating that high-quality visual images have been produced.

**Age Estimation**. In order to assess the plausibility of the images generated for a specific age group, the DEX age estimation model [18] is used to estimate the age of both the age-progressed and regressed images. The classification accuracy and 1-off classification accuracy, which measures the accuracy when the estimated age class is off by one age group from the actual age class, are the evaluation criteria used. Additionally, the Adjusted-Mean Absolute Error (MAE) is also employed as an evaluation metric, following the methodology in [25]. The MAE considers the discrepancy between the estimated age and the age range of the targeted age group. Let $\tilde{a}$ be the estimated age for a generated image that resembles the aging characteristics of an age class with range $\left[a_{l0},a_{l1}\right]$. If $\tilde{a}<a_{l0}$, Adjusted-MAE is calculated as $|a_{l0}-\tilde{a}|$. If $\tilde{a}>a_{l1}$, Adjusted-MAE is calculated as $|a_{l1}-\tilde{a}|$. Finally, if $a_{l0}<\tilde{a}<a_{l1}$, Adjusted-MAE is zero.

#### 3.4.2. Evaluation Results Using FID Score

**FID scores for the original FGNET images**. Table 1 summarizes the FID scores for the original FGNET images. The FID score is a measure of how closely the training images resemble the original FGNET images and is calculated by comparing the two sets of images. The results show that the FID scores for the images in the older age classes ($C_{5}-C_{7}$) are higher than those in the younger age classes, suggesting that the training images in the older age classes are less similar to the original FGNET images in those classes compared to the training images selected from the younger age classes and the original FGNET images in those classes.

**FID scores for images generated by CAAE**. In Table 2 the FID scores of the images generated by CAAE in age classes $C_{i}$, i=1,…7 are listed. Following the analysis in Section 3.4.1, the images with higher visual quality result in a lower FID score. The best value in each row is indicated in boldface. The lowest FID score is obtained in all age classes when the input age class is the same as the target age class ($C_{5}$) or when the input and target age classes are adjacent ($C_{1}-C_{4},C_{6}$). As can be seen, the input age class $C_{7}$ has the lowest FID score for translations to target class $C_{2}$. Since a relatively limited amount of FGNET images are contained in this age class (see Table 1), the estimated distribution for the real FGNET images that belong to class $C_{7}$ may be unrepresentative.

**FID scores for images generated by xAI-CAAE**. Table 3 and Table 4 summarize the FID scores for the generated images by xAI-CAAE for latent vectors of size 100 and θ=0.2 using Saliency and SHAP explanations, respectively. The best value in each row is indicated in boldface. Explanatory methods are used in order to more effectively assimilate the features of a facial image and provide images of higher quality than those generated by CAAE. In Table 3 and Table 4, the FID scores that outperform the original CAAE are marked with gray color to facilitate visual inspection. It is clear that when compared with the original CAAE, the xAI-CAAE using SHAP produces better FID scores for more translations than the xAI-CAAE utilizing Saliency explanations. Comparing the two xAI systems, it can be seen that the SHAP explanation method in Table 4 gives marginally improved images in some cases compared with the Saliency explanation method in Table 3. For example, all generated images by xAI-CAAE using SHAP to target age class $C_{3}$ (fourth column in Table 4) demonstrate lower FID scores compared with the images generated by xAI-CAAE using Saliency (fourth column in Table 3).

**Comparison of FID scores for different θ values**. To investigate the impact of parameter θ, another experiment is conducted by assigning a greater value to parameter θ, i.e., θ=0.5. This way, the modified gradient multiplied by the explanation matrix M is given greater weight, expecting to affect face aging more intensively. Table 5 and Table 6 summarize the FID scores for the images generated by xAI-CAAE with latent vectors of size 100 using Saliency and SHAP explanations, respectively. The best value in each row is indicated in boldface, while the FID scores that outperform those of the original CAAE are highlighted in gray. By inspecting Table 5 and Table 6, it can be seen that the proposed framework achieves lower FID scores when using SHAP than when using Saliency. More specifically, in 73.47% of the cells (36 cells out of 49) in Table 5 and Table 6, the xAI-CAAE with SHAP explanations achieved a better performance than the xAI-CAAE with Saliency explanations with respect to the FID score.

By comparing Table 3 and Table 5, it can be seen that the increase in parameter θ has lowered the FID scores in almost all cases of xAI-CAAE with Saliency explanations. Only six cells in Table 5 have greater FID scores than the corresponding cells in Table 3, i.e., cells $\left[C_{1},C_{1}\right]$, $\left[C_{7},C_{1}\right]$, $\left[C_{5},C_{4}\right]$, $\left[C_{7},C_{4}\right]$, $\left[C_{7},C_{6}\right]$, and $\left[C_{7},C_{7}\right]$. Comparing the FID scores for xAI-CAAE with SHAP explanations in Table 4 and Table 6, it is found that FID is reduced by increasing θ. FID scores for θ=0.5 in Table 6 are smaller than the FID scores for θ=0.2 in Table 4 in the vast majority of translations. Only six cells in Table 6 resulted in a larger FID than the same cells in Table 4. By inspecting the gray cells in Table 5 and Table 6, one finds that the increase in the value of θ has led to improved FID scores than those achieved by the original CAAE (see Table 2) as well as when θ=0.2. The quantitative evaluation using the FID score has demonstrated the improved performance of xAI-CAAE when explanations are incorporated from either Saliency or SHAP compared to the original CAAE without explanations. Notably, FID reduces when parameter θ increases, i.e., when a greater weight is assigned to the explanation system during training for both Saliency and SHAP. These quantitative results agree with the qualitative results depicted in Figure 5 and Figure 6 that demonstrate the visual quality of the age progressed and regressed images generated by xAI-CAAE.

**Figure 7:**
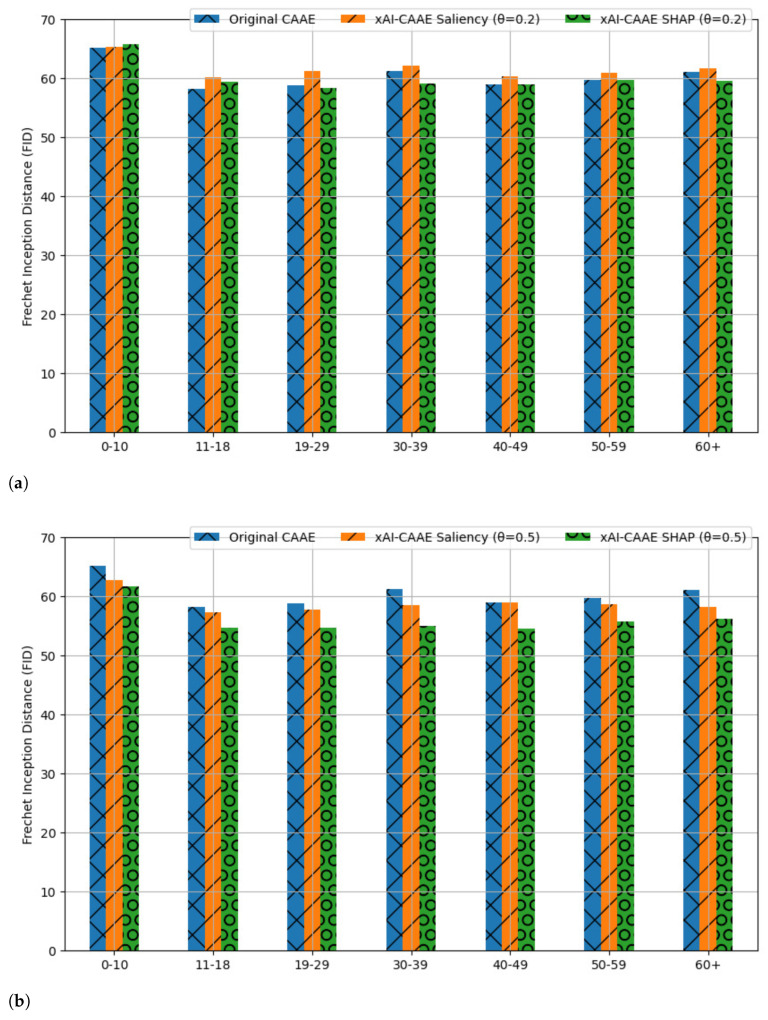
Bar diagram comparison of the FID scores in Table 7 achieved by the xAI-CAAE with latent vectors z of size 100 and (**a**) θ=0.2, (**b**) θ=0.5 against the FID scores of CAAE with latent vectors of the same size for all images generated to resemble the aging characteristics in each age class.

**Diagram comparison of FID scores for images generated by CAAE and xAI-CAAE**. To simplify the comparison of xAI-CAAE with the original CAAE, the FID score for all generated images in each age group is presented in Table 7. The distribution of the original FGNET images belonging to each age class, such as $C_{k}$, and all the generated images that resemble the aging features of this class, i.e., the images generated to age classes $C_{l}\to C_{k},l=1,\cdots ,7$, are used to calculate the FID score. Table 7 gathers FID scores for xAI-CAAE when parameter θ takes value either 0.2 or 0.5. As can be seen in Table 7, xAI-CAAE with SHAP explanation marginally outperforms CAAE for θ=0.2. When θ=0.5, xAI-CAAE with both Saliency and SHAP explanations outperforms CAAE. Notably, xAI-CAAE using SHAP achieves a large percentage of improvement in FID score compared to the original CAAE. The FID scores obtained from the calculations in Table 7 are comparatively depicted with bar diagrams in Figure 7. This representation was chosen in order to provide a visual illustration of the impact of the parameter θ on the scores. For θ=0.2, the proposed framework achieves competitive performance compared to the original CAAE (Figure 7a). For θ=0.5, xAI-CAAE with either Saliency or SHAP explanations consistently outperforms CAAE (Figure 7b), while the greatest performance gain is achieved by xAI-CAAE that employs SHAP explanations. Figure 7a,b demonstrate that increasing the value of parameter θ, which enhances the impact of the incorporated xAI technique, results in improved FID scores for the proposed xAI-CAAE, thus enabling the xAI-CAAE to surpass the original CAAE.

#### 3.4.3. Evaluation Results Using Age Estimation

**Age estimation results for images generated by CAAE and xAI-CAAE**. The pre-trained DEX age estimation model [18] was employed to determine the age estimation results of images produced by xAI-CAAE. The results of the age estimation are presented in Table 8, using the Adjusted-MAE evaluation metric described in Section 3.4.1. The fourth and fifth columns of Table 8 show the age estimation results for xAI-CAAE with latent vectors of size 100 and θ=0.2, while the last two columns display the results for the same size of latent vectors but with θ=0.5. In addition, age estimation results for images generated by CAAE are presented in the third column of Table 8, and the second column displays the evaluation metric computed for the original FGNET images. Table 8 includes results in the second column that serve as evidence of the accuracy of DEX age estimations on the 1002 FGNET images considered as ground truth. It should be noted that these results are not directly comparable to those computed for xAI-CAAE (columns 4–7) and CAAE (column 3), which were obtained for translations of the FGNET images to each of the 7 age classes resulting in a total of 7041, i.e., 1002 × 7 images per column. The evaluation of age classification accuracy and 1-off accuracy are summarized in Table 9 and Table 10, respectively.

Table 8 shows that xAI-CAAE achieves the best Adjusted-MAE for all age groups, using either Saliency or SHAP and either θ=0.2 or θ=0.5. While the differences in Adjusted-MAE between xAI-CAAE and CAAE are negligible for some age groups (such as $C_{7}$, where the top Adjusted-MAE for xAI-CAAE is 29.20 years compared to 29.64 years for CAAE), the differences are more significant for younger age groups, particularly $C_{1}$ and $C_{2}$, where xAI-CAAE achieves Adjusted-MAE scores 1.96 and 1.53 years lower than CAAE, respectively. Furthermore, xAI-CAAE using SHAP with θ=0.5 achieves the best Adjusted-MAE for all generated images (as indicated in the last row of Table 8). Hence, incorporating xAI systems in CAAE has made it easier to generate images that closely resemble the aging characteristics of the target age class.

Similar observations can be made for the age classification results in Table 9 and Table 10. The proposed framework (using either Saliency or SHAP, with either θ=0.2 or θ=0.5), achieves the top accuracy and the top 1-off accuracy for all age classes (all rows in Table 9 and Table 10). It should be noted that the most significant gain in accuracy (+5.69%) for xAI-CAAE compared to the original CAAE is achieved for age class $C_{2}$ when using SHAP with θ=0.5. As can be seen, the best results on age estimation accuracy are reported for age classes $C_{2}=\left[11,18\right]$ and $C_{3}=\left[19,29\right]$ for both CAAE and xAI-CAAE. From the results in column 2, which as mentioned are not directly comparable to the results in columns 3–7, it can be seen that the pre-trained age estimation model achieves better accuracy scores for the age classes $C_{2}=\left[11,18\right]$, $C_{3}=\left[19,29\right]$, $C_{6}=\left[50,59\right]$, and $C_{7}=60+$ on the original FGNET images. The accuracies achieved by both CAAE and xAI-CAAE for classes $C_{2}$ and $C_{3}$ are also high, but lower scores are achieved for the elder age classes $C_{6}$ and $C_{7}$. The results in column 2 for the age classes with a small number of original FGNET images (see Table 1) may not be fully representative due to the limited sample size.

Regarding 1-off accuracy, in Table 10, the most significant differences between xAI-CAAE and CAAE are noted for age classes $C_{1}$, $C_{4}$, and $C_{6}$ where the absolute differences compared to the results achieved by CAAE are 10.88%, 6.48%, and 5.59%, respectively. It can be seen that 1-off accuracy is significantly better than accuracy for all age classes, with the highest increases reported for age classes $C_{1}$ and $C_{4}$ (2752.11% and 1999.09%, respectively for xAI-CAAE with SHAP and θ=0.5). The improved performance of the images generated by xAI-CAAE on age classification illustrates the strength of xAI explanations to guide the generator for realistic age progression and regression more effectively.

**Comparison of age estimation results for different θ values**. Regarding the impact of parameter θ on xAI-CAAE, it can be seen in Table 8 that both xAI-CAAE using Saliency and SHAP achieve better Adjusted-MAE scores for θ=0.5 compared with the corresponding results for θ=0.2 for the younger age classes ($C_{1}$–$C_{4}$). The opposite is true for the older age classes ($C_{5}$–$C_{7}$) where both xAI-CAAE using Saliency and xAI-CAAE using SHAP achieved better scores for θ=0.2. It is noted that, in general, the predictions of the pre-trained age estimation model for the images generated by both xAI-CAAE and CAAE to these age classes ($C_{5}$–$C_{7}$) are less accurate, resulting in higher Adjusted-MAE scores compared with the ones achieved for age classes $C_{1}$–$C_{4}$.

### 3.5. Interpreting Age Classification Using LIME xAI-System

This experiment uses the LIME explanation system to investigate which facial features are essential for face aging. As described in detail in Section 2.2.2, LIME provides an insight into image areas (known as super-pixels) that a model trained on age classification considers critical for the classification decision. The pre-trained age classification model in [46], which is trained on the Adience dataset (see Section 3.2), is assessed using LIME. More specifically, the predictions of the pre-trained age classification model are fed to the LIME explanation system. Subsequently, LIME is used to explain the areas of the image that mainly influence the classification decision. A good classification model is expected to highlight areas of the face and not noise from the background. Since this is a fine-grained classification problem, we expect the model to highlight areas in the face that indicate each age class.

**Figure 8:**
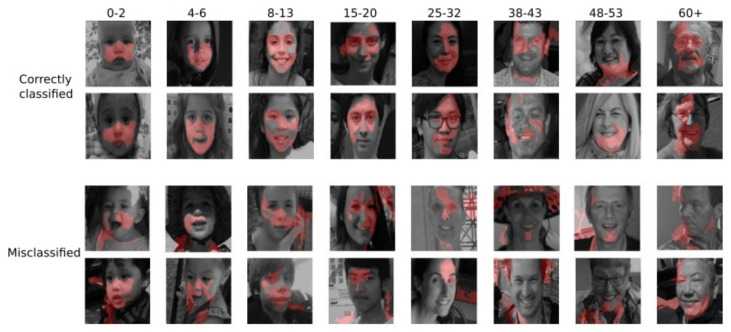
Explaining age classification predictions on Adience dataset. Two examples of correctly classified images and two misclassified images in each class are shown. Super-pixels highlight important areas of the face in each age class.

LIME provides a local interpretation of the age classification model and can be used to explain the model’s behavior for each testing image, i.e., explain the individual predictions to find out which input features are essential for the particular prediction. Figure 8 shows a subset of the experimental results, which includes two examples of correctly classified testing images and two examples of misclassified testing images in each class. It can be seen that specific areas of the face light up according to age class, indicating the facial areas that contributed the most to the classification decision. For example, the area around the cheeks is highlighted in $C_{1}$ (age 0–2), a characteristic of humans at that age. In $C_{4}$ (age 15–20), the model identifies the area of the eyes as important, while the neck area is highlighted in $C_{7}$ (age 48–53). For the misclassified images, we notice that the same regions are highlighted, but the noise from the background is also considered important. This experiment demonstrates that by leveraging LIME in the training process of an age classifier, the classifier’s attention can be directed toward the facial areas that play a crucial role in accurate classification decisions. Additionally, considering the significant facial areas of misclassified images can help the classifier reduce its attention to these areas and improve the accuracy of age classification.

## 4. Discussion and Conclusions

In this paper, a novel explainable Conditional Adversarial Autoencoder, termed xAI-CAAE, aims to provide corrective feedback from the discriminator to the generator through an explanation matrix using Saliency maps and Shapley values as explanatory techniques. The proposed framework has been thoroughly evaluated both qualitatively and quantitatively. It has been demonstrated to have great potential as a competitive framework for generating more realistic face images. The xAI system has contributed significantly to face aging, as can be confirmed by quantitative evaluation metrics, such as the FID scores and the age estimation on the generated images. LIME has also been leveraged to investigate the facial areas important for age classification, yielding interesting results. Future work will focus on deepening the xAI methods for face aging.

## Figures and Tables

**Table 1:** FID scores for the original FGNET images across age classes $C_{i}$, i=1,…,7. The number of images that belong to each age class is listed in the second column.

Age Class	Number of Images	FID Score
$C_{1}=\left[0,10\right]$	411	76.81
$C_{2}=\left[11,18\right]$	276	61.69
$C_{3}=\left[19,29\right]$	167	68.03
$C_{4}=\left[30,39\right]$	79	87.53
$C_{5}=\left[40,49\right]$	46	104.21
$C_{6}=\left[50,59\right]$	15	141.61
$C_{7}=\left[60,80\right]$	8	162.13

**Table 2:** FID scores for the images generated by CAAE for the size of latent vectors z equal to 100 in age classes $C_{i},i=1,2,\ldots ,7$. The best value in each row is indicated in boldface.

Input	Target Age Class						
**Age Class**	$C_{1}$	$C_{2}$	$C_{3}$	$C_{4}$	$C_{5}$	$C_{6}$	$C_{7}$
$C_{1}$	93.51	**77.30**	78.50	80.83	78.63	79.71	80.49
$C_{2}$	79.96	77.50	**76.58**	80.77	76.92	77.93	79.04
$C_{3}$	83.57	**74.71**	78.58	77.54	76.09	75.42	77.43
$C_{4}$	97.57	92.40	91.86	90.06	**89.42**	94.41	91.21
$C_{5}$	115.48	108.74	105.38	105.82	**103.31**	109.18	108.22
$C_{6}$	137.41	129.38	132.62	127.72	126.99	127.10	**124.24**
$C_{7}$	145.50	**131.44**	143.80	137.37	134.87	132.69	137.51

**Table 3:** FID scores for images generated by the proposed xAI-CAAE for latent vectors z of size 100 and θ=0.2 using Saliency explanation in age classes $C_{i},i=1,\ldots ,7$. The best value in each row is indicated in boldface. The FID scores that outperform those of the original CAAE (Table 2) are highlighted in gray.

Input	Target Age Class						
**Age Class**	$C_{1}$	$C_{2}$	$C_{3}$	$C_{4}$	$C_{5}$	$C_{6}$	$C_{7}$
$C_{1}$	88.44	**76.81**	79.77	79.73	77.61	77.93	79.62
$C_{2}$	83.91	81.68	81.47	81.12	79.80	80.16	**78.72**
$C_{3}$	83.16	82.64	83.09	83.15	**81.47**	82.02	83.61
$C_{4}$	103.90	92.46	94.31	100.82	94.27	**91.95**	96.96
$C_{5}$	119.68	107.53	112.02	112.97	113.55	**105.92**	113.72
$C_{6}$	153.28	132.22	142.72	**123.51**	136.57	139.22	139.33
$C_{7}$	134.93	133.58	136.56	**120.42**	128.27	133.16	136.79

**Table 4:** FID scores for images generated by the proposed xAI-CAAE for latent vectors z of size 100 and θ=0.2 using SHAP explanation in age classes $C_{i},i=1,\ldots ,7$. The best value in each row is indicated in boldface. The FID scores that outperform those of the original CAAE (Table 2) are highlighted in gray.

Input	Target Age Class						
**Age Class**	$C_{1}$	$C_{2}$	$C_{3}$	$C_{4}$	$C_{5}$	$C_{6}$	$C_{7}$
$C_{1}$	91.76	75.31	**72.07**	77.33	75.06	78.38	76.11
$C_{2}$	85.24	**78.74**	79.19	79.38	79.12	79.16	79.17
$C_{3}$	82.59	80.25	77.81	**74.43**	76.85	77.73	77.74
$C_{4}$	101.42	**89.87**	91.11	92.76	92.86	92.01	94.62
$C_{5}$	119.23	**106.70**	108.25	107.91	109.82	112.48	111.47
$C_{6}$	145.47	**116.81**	121.69	121.18	126.64	130.71	127.28
$C_{7}$	137.68	141.90	134.83	145.01	133.76	**129.72**	146.28

**Table 5:** FID scores for images generated by the proposed xAI-CAAE for latent vectors z of size 100 and θ=0.5 using Saliency explanation for age classes $C_{i},i=1,\ldots ,7$. The best value in each row is indicated in boldface. The FID scores that outperform the original CAAE (in Table 2) are highlighted in gray.

Input	Target Age Class						
**Age Class**	$C_{1}$	$C_{2}$	$C_{3}$	$C_{4}$	$C_{5}$	$C_{6}$	$C_{7}$
$C_{1}$	89.13	74.18	**73.19**	76.73	77.11	77.10	75.58
$C_{2}$	81.34	76.53	79.04	79.40	76.61	77.23	**75.80**
$C_{3}$	80.04	81.52	78.92	**75.37**	77.52	76.44	77.20
$C_{4}$	94.75	87.55	89.68	**86.18**	89.43	89.89	88.95
$C_{5}$	114.29	105.91	101.32	118.98	112.86	**100.82**	110.00
$C_{6}$	141.69	117.76	120.19	**116.91**	126.94	118.87	125.98
$C_{7}$	137.04	**122.92**	135.70	123.84	127.32	134.07	149.07

**Table 6:** FID scores for images generated by the proposed xAI-CAAE for latent vectors z of size 100 and θ=0.5 using SHAP explanation for age classes $C_{i},i=1,\ldots ,7$. The best value in each row is indicated in boldface. The FID scores that outperform the original CAAE (in Table 2) are highlighted in gray.

Input	Target Age Class						
**Age Class**	$C_{1}$	$C_{2}$	$C_{3}$	$C_{4}$	$C_{5}$	$C_{6}$	$C_{7}$
$C_{1}$	89.49	70.90	69.95	70.22	**69.25**	73.65	72.88
$C_{2}$	76.00	74.77	73.08	74.26	**72.86**	74.79	74.50
$C_{3}$	79.17	76.56	76.54	73.36	74.71	73.33	**72.67**
$C_{4}$	93.85	87.22	**86.05**	86.58	89.39	88.05	90.12
$C_{5}$	112.37	**101.81**	109.32	107.68	109.64	107.66	106.52
$C_{6}$	135.24	134.39	**117.76**	126.24	152.73	118.30	142.22
$C_{7}$	131.26	136.74	134.48	**128.89**	144.58	142.89	135.19

**Table 7:** FID scores for the images generated by xAI-CAAE and CAAE for latent vectors z of size 100 in age classes $C_{i},i=1,\ldots ,7$. The best value in each row is indicated in boldface.

Age Class	CAAE	xAI-CAAE (θ=0.2)		xAI-CAAE (θ=0.5)	
		Saliency	SHAP	Saliency	SHAP
$C_{k}\to C_{1},k=1,\cdots ,7$	65.16	65.37	65.76	62.79	**61.69**
$C_{k}\to C_{2},k=1,\cdots ,7$	58.16	60.20	59.45	57.39	**54.67**
$C_{k}\to C_{3},k=1,\cdots ,7$	58.78	61.20	58.31	57.77	**54.75**
$C_{k}\to C_{4},k=1,\cdots ,7$	61.18	62.18	59.18	58.51	**54.97**
$C_{k}\to C_{5},k=1,\cdots ,7$	58.97	60.39	58.96	58.92	**54.55**
$C_{k}\to C_{6},k=1,\cdots ,7$	59.75	60.99	59.69	58.71	**55.79**
$C_{k}\to C_{7},k=1,\cdots ,7$	61.04	61.74	59.54	58.24	**56.24**

**Table 8:** Adjusted-MAE in age estimation for the proposed xAI-CAAE using Saliency and SHAP explanations. Age prediction is performed using the pre-trained model in [18]. Adjusted-MAE for the original FGNET images and the images generated by the original CAAE are also listed. The best value in each row (excluding column 2) is indicated in boldface. The results in the second column prove the correctness of the DEX age estimations on the ground truth FGNET images and cannot directly be compared to the results listed in the remaining columns.

	Adjusted-MAE					
Age Class	FGNET	CAAE	xAI-CAAE (θ=0.2)		xAI-CAAE (θ=0.5)	
			Saliency	SHAP	Saliency	SHAP
$C_{1}$	13.03	12.99	15.29	11.41	12.93	**11.03**
$C_{2}$	5.83	5.65	5.98	4.56	5.31	**4.12**
$C_{3}$	5.81	3.08	3.94	3.09	**2.30**	2.74
$C_{4}$	10.94	9.30	10.01	9.60	**8.76**	9.47
$C_{5}$	9.15	16.08	16.19	**15.90**	16.34	16.91
$C_{6}$	0.20	22.56	**21.54**	22.83	22.44	23.96
$C_{7}$	10.13	29.64	**29.20**	30.29	30.64	31.01
$C_{k},k=1,\cdots ,7$	18.05	17.45	18.37	17.46	17.15	**16.90**

**Table 9:** Accuracy of age classification for the proposed xAI-CAAE using Saliency and SHAP explanations. Age prediction is performed using the pre-trained model in [18]. Accuracy for the original FGNET images and the images generated by the original CAAE are also listed. The best value in each row (excluding column 2) is indicated in boldface. The results in column 2 prove the correctness of the DEX age estimations on the ground truth FGNET images and cannot directly be compared to the results in the remaining columns.

	Accuracy (%)					
Age Class	FGNET	CAAE	**xAI-CAAE (θ=0.2)**		**xAI-CAAE (θ=0.5)**	
			Saliency	SHAP	Saliency	SHAP
$C_{1}$	10.22	1.70	**3.99**	2.99	3.39	1.90
$C_{2}$	38.41	35.03	34.33	39.02	34.43	**40.72**
$C_{3}$	52.69	59.68	55.99	54.39	**61.48**	59.68
$C_{4}$	8.86	4.29	2.69	4.89	**5.19**	3.29
$C_{5}$	4.35	3.29	2.59	2.99	**3.69**	2.89
$C_{6}$	26.67	5.99	**7.88**	6.89	6.79	5.79
$C_{7}$	75.00	10.08	**13.37**	9.58	9.18	10.88
$C_{k},k=1,\cdots ,7$	25.45	17.15	17.27	17.25	17.74	**17.88**

**Table 10:** Results for 1-off accuracy of age classification for the proposed xAI-CAAE using Saliency and SHAP explanations. Age prediction is performed using the pre-trained model in [18]. The 1-off accuracy scores of age classification for the original FGNET images and the images generated by the original CAAE are also listed. The best value in each row (excluding column 2) is indicated in boldface. The results in column 2 provide proof of the correctness of the DEX age estimations on the ground truth FGNET images and cannot directly be compared to the results in the remaining columns.

	1-Off Accuracy					
Age Class	FGNET	CAAE	**xAI-CAAE (θ=0.2)**		**xAI-CAAE (θ=0.5)**	
			Saliency	SHAP	Saliency	SHAP
$C_{1}$	52.80	45.01	43.71	**55.89**	51.00	54.19
$C_{2}$	88.04	89.02	87.13	91.02	89.72	**92.71**
$C_{3}$	80.84	90.22	87.82	91.02	**92.81**	91.42
$C_{4}$	48.10	66.37	64.97	64.37	**72.85**	69.06
$C_{5}$	54.35	12.48	15.57	**16.07**	13.07	11.18
$C_{6}$	100	18.26	**23.85**	19.26	18.86	15.97
$C_{7}$	75.00	19.66	**22.06**	18.06	16.77	17.96
$C_{k},k=1,\cdots ,7$	67.76	48.72	49.30	**50.81**	50.73	50.36

## Data Availability

All data showcased in this article are available for download and further analysis through their respective websites. Any additional information or clarifications on the data used in this study can be obtained by contacting the corresponding author.
